# Infrared Thermography Measurement for Vibration-Based Structural Health Monitoring in Low-Visibility Harsh Environments

**DOI:** 10.3390/s20247067

**Published:** 2020-12-10

**Authors:** Jia-Hao He, Ding-Peng Liu, Cheng-Hsien Chung, Hsin-Haou Huang

**Affiliations:** 1 Department of Engineering Science and Ocean Engineering, National Taiwan University, Taipei 10617, Taiwan; r04525079@g.ntu.edu.tw; 2 Ship and Ocean Industries R&D Center, New Taipei City 251401, Taiwan; calvinliu@mail.soic.org.tw (D.-P.L.); chcs@mail.soic.org.tw (C.-H.C.)

**Keywords:** structural health monitoring, infrared thermal imager, harsh environment, modal analysis, damage identification, mode shape recombination

## Abstract

In this study, infrared thermography is used for vibration-based structural health monitoring (SHM). Heat sources are employed as sensors. An acrylic frame structure was experimentally investigated using the heat sources as structural marker points to record the vibration response. The effectiveness of the infrared thermography measurement system was verified by comparing the results obtained using an infrared thermal imager with those obtained using accelerometers. The average error in natural frequency was between only 0.64% and 3.84%. To guarantee the applicability of the system, this study employed the mode shape curvature method to locate damage on a structure under harsh environments, for instance, in dark, hindered, and hazy conditions. Moreover, we propose the mode shape recombination method (MSRM) to realize large-scale structural measurement. The partial mode shapes of the 3D frame structure are combined using the MSRM to obtain the entire mode shape with a satisfactory model assurance criterion. Experimental results confirmed the feasibility of using heat sources as sensors and indicated that the proposed methods are suitable for overcoming the numerous inherent limitations associated with SHM in harsh or remote environments as well as the limitations associated with the SHM of large-scale structures.

## 1. Introduction

Currently, traditional sensors [1,2,3,4], including accelerometers, strain gauges, capacitance sensors, fiber Bragg grating sensors and so on, are used for structural health monitoring (SHM); however, these sensors are expensive, take a long time to install, and tend to wear out. These sensors may also damage or get entangled between transmission lines [5]; in addition, their accuracy depends on the humidity [6], and they require light of a sufficient amplitude to function [7]. The cumbersomeness of sensors inconveniences construction workers and can cause fatal injuries. Poozesh et al. [8] reported that a structural monitoring system for a 50-m-long wind turbine blade requires 200 strain gauges, costing between USD 35,000 and 50,000; moreover, three weeks are required for installing these gauges, and more sensors are required for monitoring the entire area. Contact sensors require transmission lines to transmit signals to the data calculation system, and these lines may cause difficulties in repair and affect the structure itself.

To overcome these problems, some scholars have proposed SHM methods using image-based measurement techniques [9,10,11,12,13,14,15,16,17]. Image-based measurement methods have been widely used in various experimental mechanics studies. Compared with measurement using traditional sensors, image-based measurement is a noncontact measurement technology that does not interfere with the test object. Image-based measurement can be divided according to the usage of marked [9,18,19,20] or unmarked points [21,22,23]. Measurement with marked points involves attaching a specific pattern to a structure for obtaining a strong contrast between the structure and background for acquiring a clear image. Conversely, in measurement with unmarked points, there is no need to attach a specific pattern to a structure because the structure already has certain features, such as color or unique signs, which can function as marked points; thus, there is already considerable contrast between the structure and background. Image-based measurement is used to obtain the position information and calculate the displacement of a structure. High-speed cameras have proven to be highly reliable in identifying modal parameters. However, these cameras require a bright light source. Currently, most researchers employ additional light sources when using high-speed cameras [24,25,26,27]. However, the light is usually insufficient or nonhomogeneous under two conditions: when the equivalent measurement distance is long, and when the object to be measured is large. Therefore, this study proposed a measurement method using an infrared thermal imager to improve measurement under that harsh environments.

Infrared thermal imaging is widely employed in civil engineering and construction, nondestructive testing of composite materials, monitoring and maintenance, vibrothermography (sonic infrared), fluid dynamics and energetics, microscale applications, thermodynamics, the environmental engineering field, thermal imaging systems, biomedical applications, and thermophysics [28]. Nondestructive testing is a highly valuable technique that can simultaneously reduce cost and save time in product evaluation, troubleshooting and research because it does not alter the targeted objects during the inspection. It has a wide variety of applications such as power systems [29], aerospace [30] and robotics [31]. Noncontact testing is gaining prominence for nondestructive testing due to its feasibility without physical contact. For example, in sonic infrared, ultrasonic or high-frequency excitation is used to vibrate a structure, increasing the temperature of cracks. Then, the crack and internal damage are traced [32,33]. Scholars have used infrared thermal imaging cameras for modal analysis in recent years. An infrared thermal imager tracks the temperature signal of a structure and then inverts the characteristics of the structure, such as the natural frequency or displacement signal [34,35,36].

SHM with traditional sensors is expensive and requires complex wiring. Moreover, considerable time must be spent on installing the sensors. The image-based measurement system, a new type of measurement method, has been recently used for SHM. Although it helps overcome the problems associated with traditional sensors, the image-based measurement system cannot measure structures accurately under low visibility. Ye et al. [37] attempted to apply a charge-coupled device (CCD) camera in vision-based measurement and found that it was not suitable for use under dark or hazy conditions. Therefore, this study proposes an infrared thermography measurement system to replace the conventional image-based measurement system to overcome this problem. The proposed method is suitable for conducting measurements in low-visibility environments.

The article is organized as follows: The referred and the proposed methods used for infrared thermography measurements and data processing are described in Section 2. Validation of the feasibility of the proposed infrared thermography measurement and comparison of the measurements under various conditions are presented in Section 3. When facing a large structure, the measuring distance and image resolution become issues. Section 4 demonstrates a proposed method to overcome the aforementioned challenge. Moreover, the proposed method is extended to the application of damage identification under harsh environments, and the results are presented in Section 5. Last, conclusions of the present study are made, and potential future works are discussed in Section 6.

## 2. Methodology of Infrared Thermography Measurement and Data Processing

### 2.1. Sensing Device for Measurements

An infrared thermal imager with an accuracy of 2 °C, spatial resolution of 1.38 mrad, and frame rate of 60 Hz (FLIR T420, FLIR Systems, Wilsonville, OR, USA) was used for infrared thermography measurements. Heat sources (TSA(C)013d000bR29.7; Taiwan KLC PTC Co. Ltd., Taichung, Taiwan) were attached to the tested structure for capturing vibration responses. The outer diameter, reference temperature rise, power density, power, resistance, and current of the heat source were 13 mm, 30 °C, 0.23 W/cm^2^, 0.3 W, 29.7 Ω, and 0.1 A, respectively.

### 2.2. Image Processing

In this study, an infrared thermal imaging device was used for measurement, data capture, and data preprocessing. When the background is cluttered and the surrounding interference is high, the temperature span of the image can be adjusted to enable easy identification of the target.

After an image is imported into MATLAB, it is grayscaled and binarized using the MATLAB built-in function, which employed Otsu’s method [38] to choose the threshold for minimizing the intraclass variance of the black and white pixels. The function computes a global threshold that can be used to convert an intensity image to a binary image. Due to the strong temperature contrast between heat sources and background, the noise in the image can be removed to obtain the structural response. Particularly during the tests of the present study, the thermal range of the imager was set between 26 and 35 °C, while room temperature is around 25 °C and the temperature of heat resource is 30 °C. After converting a color image into a grayscale image, each pixel has a grayscale value. A global threshold established using the aforementioned function can be effective for binarization due to strong contrast of temperature in the vicinity of the heat sources area, and, therefore, the noise near the heat sources is eliminated. Then, we crop the images, remaining only the heat sources area to remove noise far from the heat sources. In addition, owing to the uneven heat sources, voids typically appear inside the region of heat sources during binarization, and, therefore, 8-connectivity [39] is employed to repair the defects. If two neighboring pixels connected in transverse, longitudinal and diagonal directions, they are treated as a region. Every pixel inside the region with value 0 is filled to mark the entire region of the heat source in the 2D image. After obtaining the characteristics of the target, the displacement data of the target in each photograph are obtained according to the center of the heat source region.

### 2.3. Frequency Domain Decomposition (FDD)

In this study, the modal parameters are calculated using the FDD method in the operational modal analysis, and the power spectrum density (PSD) of the output is subjected to singular value decomposition (SVD) to decompose the PSD into a set of single-degree-of-freedom (SDOF) systems corresponding to multiorder modes. Details of the theoretical background of FDD are referred to [40,41,42]. Brief introduction is as follows:

First, the relationship between the unknown input signal *x*(t) and measured response signal *y*(t) is expressed using the following equation:

(1) $$\left[G_{yy}\left(\omega \right)\right]=\left[\bar{H}\left(\omega \right)\right]\left[G_{xx}\right]{\left[H(\omega )\right]}^{T},$$

where $G_{xx}$ is the (*r* × *r*) input PSD matrix, *r* is the number of inputs, $G_{yy}$ is the (*m* × *m*) output PSD matrix of the response, and m is the number of responses. *H*(*ω*) is the *m* × *r* frequency response function matrix, and the overline and superscript T denote the complex conjugate and transpose, respectively. Assuming that the input is white noise and, in the case of weak damping, the output response PSD matrix is rewritten as Equation (2) and the modality is decomposed:

(2) $$G_{yy}\left(\omega \right)=\sum_{k\in Sub\left(\omega \right)}\frac{d_{k}\phi_{k}\phi_{k}^{T}}{j\omega -\lambda_{k}}+\frac{{\bar{d}}_{k}{\bar{\phi }}_{k}{\bar{\phi }}_{k}^{T}}{j\omega -\lambda_{k}}.$$

where $d_{k}$ is a scalar constant. In FDD, the PSD matrix is predicted first; the sensor signal is composed of $G_{yy}\left(\omega \right)$. Then, SVD is performed, as shown in Equation (3), to obtain a singular value spectrum map in which the peaks indicate the natural frequencies of the various modes.

(3) $${\hat{G}}_{yy}\left(\omega_{i}\right)=U_{i}S_{i}U_{i}^{H},$$

where $U_{i}=\left[u_{i1},u_{i2},u_{i3},\ldots ,u_{im}\right]$ is a positive matrix composed of singular vectors, $S_{i}$ is a diagonal matrix composed of scalar singular values, and the mode shape of the first mode is $u_{i1}$. This SVD method identifies dense and adjacent modalities with high accuracy and can resist noise. It is extremely popular in engineering applications.

### 2.4. Modal Assurance Criterion

To verify the results obtained using the thermal imager, we quantify the difference between the mode shape determined through the thermal imaging device and the mode shape determined through accelerometers by using the modal assurance criterion (MAC). Mode shapes must satisfy orthogonality. Modes of different orders must be orthogonal to each other, and the MAC must be 0. The mode shapes calculated using the same mode must satisfy the dependence, so the diagonal MAC must be close to 1.

The MAC formula [43] is as follows:

(4) $$MAC_{rq}=\frac{{\left|{\left\{\phi_{A}\right\}}_{r}^{T}{\left\{\phi_{B}\right\}}_{q}\right|}^{2}}{\left({\left\{\phi_{A}\right\}}_{r}^{T}{\left\{\phi_{A}\right\}}_{r}\right)\left({\left\{\phi_{B}\right\}}_{q}^{T}{\left\{\phi_{B}\right\}}_{q}\right)},$$

where $MAC_{rq}$ is the rth row and qth column element of the *MAC* matrix. In this study, $\phi_{A}$ represents the mode shape calculated using the accelerometer, and $\phi_{B}$ is the mode shape calculated using the thermal imager. The *MAC* value represents the inner product between the different modal shape vectors and is between 0 and 1. If the *MAC* is 0, the intersection angle of the mode shape vector is 90°, indicating that the correlation between modes is weak. Conversely, if the *MAC* is close to 1, the correlation between modes is strong; a value between 0.95 and 1.00 is assumed to be acceptable for all practical purposes [44,45].

### 2.5. Mode Shape Curvature Method (MSCM)

The MSCM is the second derivative of the mode shape, and its relationship with the stiffness of the structure is as follows [46]:

(5) $$\phi^{\prime \prime }\left(x\right)=\frac{M\left(x\right)}{EI},$$

where ϕ″(*x*) is the mode shape curvature at point *x*, M(x) is the bending moment, and *EI* is the flexural rigidity. Therefore, when the structure is damaged, the Young’s modulus decreases and the mode shape curvature increases. The MSCM then distinguishes between damaged and undamaged structures from the curvature difference between two neighboring nodes. The relationship is as follows:

(6) $$\Delta \phi_{i}^{\prime \prime }=\left|\phi_{i,u}{}^{\prime \prime }-\phi_{i,d}{}^{\prime \prime }\right|$$

where $\phi_{i}{}^{\prime \prime }\left(x\right)$ represents the shape curvature of the *i*th mode, and *u* and *d* represent an undamaged structure and damaged structure, respectively. When the structure is locally damaged, a reduction in the local structural stiffness occurs, which results in local curvature differences; therefore, the curvature is defined using a central difference equation:

(7) $$\phi_{i,j}{}^{\prime \prime }=\frac{\phi_{i+1,j}-2\phi_{i,j}+\phi_{i-1,j}}{h^{2}},$$

where h is the distance between nodes, and $\phi_{i,j}$ is the *i*th mode under the *j*th coordinate.

### 2.6. Proposed Mode Shape Recombination Method (MSRM)

Currently, the resolution of images captured by high-speed cameras used for SHM typically ranges from 640 × 480 pixels to 1400 × 1024 pixels [47]. The low resolution of imagers is due to the limitations imposed by national policy on commercial infrared thermal imaging. Therefore, insufficient resolution limits measurement over a short distance. However, for large structures, such as offshore wind turbines, dynamic response measurement must be performed at a remote location to capture the entire structure. To increase the image resolution, the imager must be close to the structure to identify the mode shape of the structure. However, this would lead to only a partial mode shape of the entire structure being obtained. Therefore, we employ the MSRM for facilitating the measurement of large structures. A large-scale structure is measured using image stitching, and at least two heat sources are captured in one image.

For instance, the first image contains the first and second heat sources, the second image contains the second and third heat sources, the third image contains the third and fourth heat sources, and so on. After the image-distortion-correction process, the overlapped measurement datasets are employed to normalize the partial mode shapes to a suitable direction and magnitude, after which all normalized datasets are combined into a complete mode shape. The MSRM overcomes the problem that infrared thermal imagers cannot measure all the heat sources in large-scale structures simultaneously.

**Figure 1:**
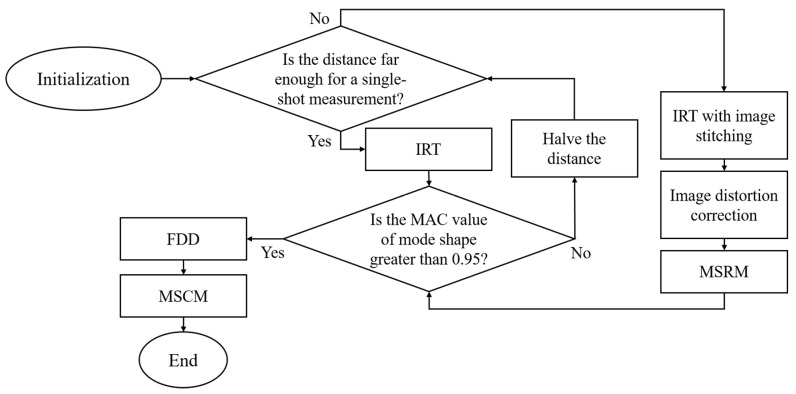
Flowchart of the infrared thermography measurement.

A flowchart of the infrared thermography measurement used for SHM is shown as Figure 1. The optimal distance between the imager and structure is determined to adapt to various situations.

## 3. Infrared Thermography Measurement for SHM in Normal and Harsh Environments

### 3.1. Experimental Setup

**Figure 2:**
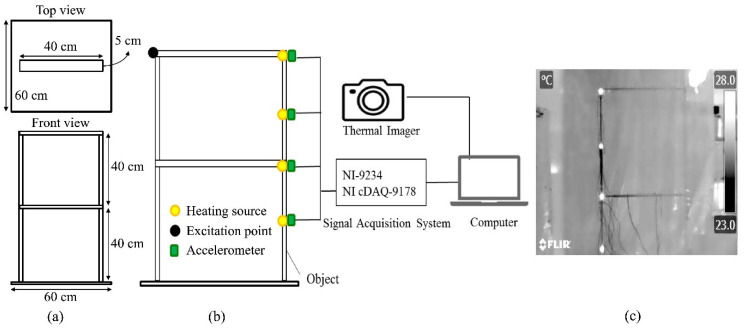
(**a**) Acrylic frame structure, (**b**) experimental setup, and (**c**) a sample image of the physical model through the thermal imager.

The main objective of this experiment was to verify whether infrared thermography is suitable for SHM under harsh environmental conditions. An acrylic frame structure [46] was used as the test specimen; it consisted of six 40 cm × 5 cm × 0.5 cm acrylic slats that were connected by angle steel at the corners. The structure was mounted on a 60 cm × 60 cm × 0.5 cm acrylic plate, as shown in Figure 2a. The test frame was excited using an impact hammer (PCB-086C03, PCB Piezotronics, Depew, NY, USA). The infrared thermal imager was placed in front of the frame and connected to a computer so that the vibration responses from the heat sources were captured. The heat sources, linearly placed with 20 cm spacing for the adjacent ones, were adhered to the structure and employed as the target of measurement under room temperature (25 °C; Figure 2b). The resolution, measurable temperature range, frame rate, and minimum focal length of the infrared thermal imager were 320 × 240 pixels, −20 to 650 °C, 30 Hz, and 40 cm, respectively.

For comparison, the vibration responses were simultaneously measured by four accelerometers with a sensitivity of 10.2 mV/(m/s^2^) ± 10%, the peak value of measurement range ±491 m/s^2^ and the root mean square (rms) value of broadband resolution 0.0015 m/s^2^ (PCB-352C65, PCB Piezotronics, Depew, NY, USA). The accelerometers were connected to a dynamic signal acquisition module (NI-9234, National Instruments, Austin, TX, USA) with a noise of 50 μVrms at the maximum sample rate and chassis (NI cDAQ-9178, National Instruments, Austin, TX, USA).

We conducted several experiments in four environmental conditions (namely, Normal, Dark, Hindered, and Hazy environments) and compared the results obtained using the accelerometers with those obtained using the infrared thermal imager to verify the feasibility of using the infrared thermal imager for measurement. Specifically, the experiment performed with the infrared thermal imager under four environmental conditions was conducted three times each. In total, 12 experimental data measured using accelerometers were collected.

### 3.2. Measurement with Frame Structure under Normal Conditions

**Figure 3:**
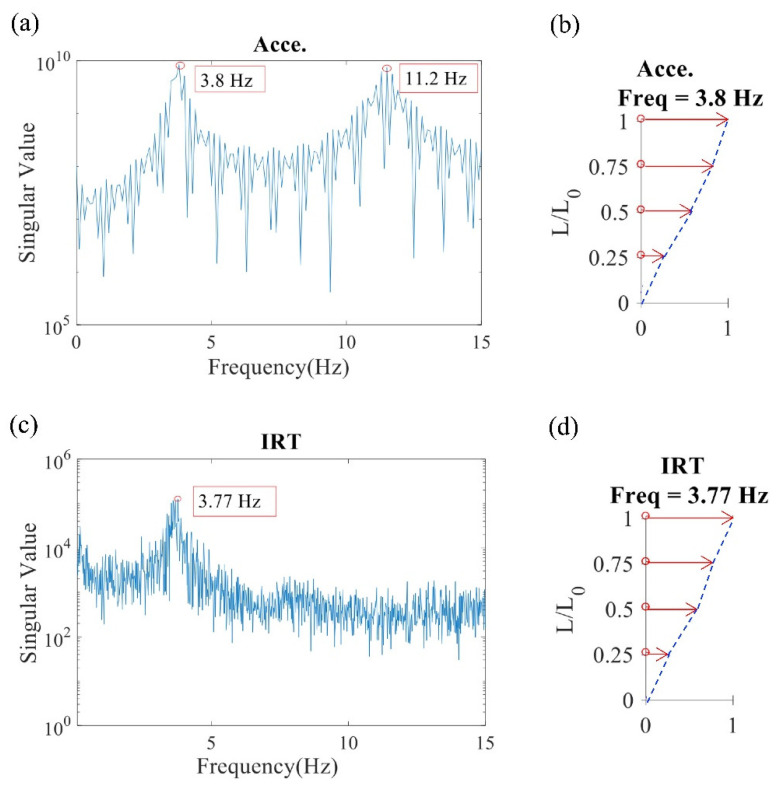
Singular value spectrums and mode shapes: (**a**) natural frequency obtained through the accelerometer measurement, (**b**) corresponding mode shapes obtained through the accelerometer measurement, (**c**) natural frequency obtained through the infrared thermography measurement, and (**d**) corresponding mode shape obtained through the infrared thermography measurement. (Acce.: accelerometers; IRT: infrared thermography; L_0_ = 80 cm).

The acceleration signals of the frame were processed using modal analysis to obtain the dynamic response of the structure. The natural frequency of the structure was determined according to the singular value spectrum. Let the first and second natural frequencies be denoted $f_{1}$ and $f_{2}$, respectively. As shown in the singular value spectrum, the first and second natural frequencies obtained using the accelerometers ($f_{1}^{Acce}$ and $f_{2}^{Acce}$, respectively) were 3.8 and 11.2 Hz, respectively (Figure 3a). The corresponding mode shape of the first mode is displayed in Figure 3b. Modal analysis was performed on the data obtained using the infrared thermal imager. The relative displacement of the frame was obtained using the image processing (discussed in Section 2.1), after which the corresponding singular value spectrum was obtained (Figure 3c). As illustrated in Figure 3c, the first natural frequency ($f_{1}^{IRT})$ was 3.77 Hz, and the corresponding mode shape is shown in Figure 3d.

The maximum, average, minimum, and standard deviation values of the calculated first natural frequencies of the infrared thermography (IRT) and accelerometers (Acce.) results are listed in Table 1. The values of IRT and Acce. results for four environments were calculated separately using each of the three experimental data. The error is defined as the absolute value of the difference between the two average natural frequencies ($\left|\mathrm{Average}\;f_{1}^{IRT}-\mathrm{Average}\;f_{1}^{Acce}\right|$) divided by the average natural frequency obtained through the accelerometer measurement ($\mathrm{Average}\;f_{1}^{Acce}$); the error was 1.35%. A possible reason for this error is the time and spatial resolution of the different measurements. However, the average MAC of the first mode shapes obtained through the accelerometer and infrared thermal imager measurement methods was 0.9963, indicating that the results obtained using the two measurement methods were in favorable agreement. The standard deviation among the three measurements is only 0.11, therefore, the thermal imager is proved to be applicable. Unfortunately, the infrared thermal imager could not identify the second mode due to the lack of spatial resolution. This limitation can be overcome by using high-end devices.

### 3.3. Measurement with Frame Structure under Harsh Environments

**Figure 4:**
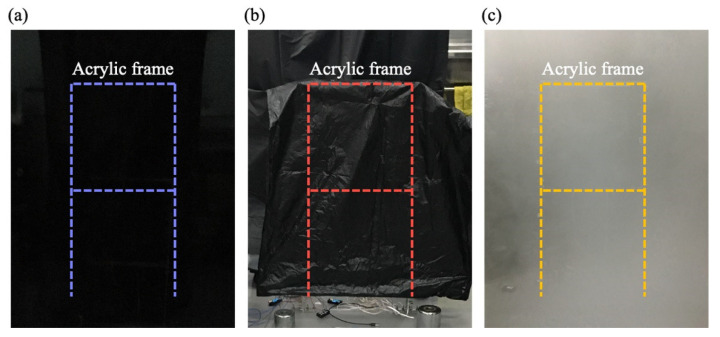
Experiment layouts for artificially (**a**) dark, (**b**) hindered, and (**c**) hazy environments.

The experimental layouts for the artificially dark, hindered, and hazy environments are shown in Figure 4a–c, respectively.

#### 3.3.1. Dark Environment

**Figure 5:**
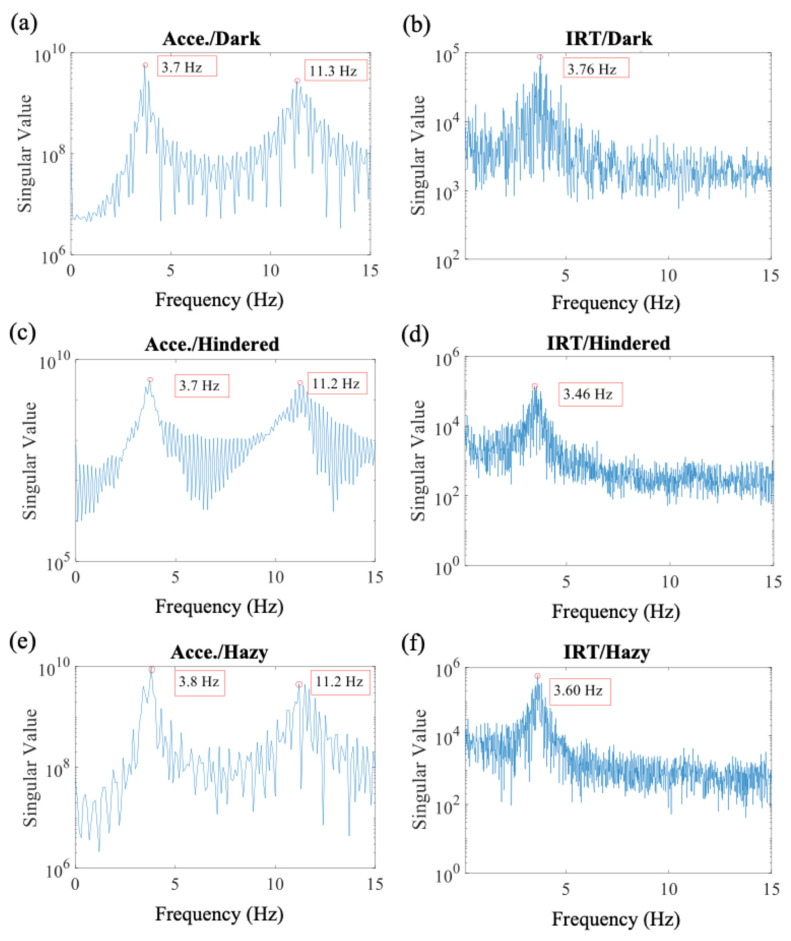
Natural frequency results in three environments: (**a**) the thermal imager in a dark environment, (**b**) accelerometers in a dark environment, (**c**) the thermal imager in a hindered environment, (**d**) accelerometers in a hindered environment, (**e**) the thermal imager in a hazy environment, and (**f**) accelerometers in a hazy environment. (Acce.: accelerometers; IRT: infrared thermography).

Theoretically speaking, the performance of the thermal imager is not dependent on the lighting conditions. The measurement using the infrared thermal imager could be performed even with insufficient light because of the imaging principle; if we were to use general optical cameras to perform the measurement under low-visibility conditions, we would require additional illumination devices, such as light-emitting diodes (LEDs). Such a solution is infeasible in a completely dark and remote environment. We conducted infrared thermography measurements in an artificially dark environment (Figure 4a). Three measurements were performed with the infrared thermal imager, and three datasets were obtained for data processing. One of the measured datasets is shown in Figure 5a,b. The average values of the first natural frequencies are listed in Table 1.

Figure 5a,b indicate that the thermal imager measurements were in favorable agreement with the accelerometer measurements. The error for the two average first natural frequencies was only 0.64%. The mode shape obtained from thermal measurement was highly consistent with that obtained from accelerometer measurement, and the average MAC was 0.9976 (Table 1). The results indicate that the thermal imager is suitable for use in measurements performed in dark environments; the performance of the thermal imager was better than that of the optical camera, with which measurements have to be performed under sufficient light.

#### 3.3.2. Hindered Environment

Manmade obstacles, such as protection covers, and natural obstacles, such as dust, can hinder measurements. Clearing or removing natural obstacles is expensive, and costs are higher when performing measurements in a harsh or remote environment. To simulate interference due to obstacles, we completely covered the structure with a black plastic bag (Figure 4b). Examples of the measured datasets are shown in Figure 5c,d. The error between the two average first natural frequencies was 3.12%, and the average MAC was 0.9808 (Table 1). This implies that there might be a deviation of frequency values between the two different sensors. Nevertheless, the low standard deviation of data from the thermal imager shows acceptable reliability of the thermal imager measurement under a hindered environment. Although the thin plastic bag might hinder the radiation of heat slightly, the vibration signal can still be identified. The results indicate that despite the slight interference caused by the plastic bag, the thermal imaging device is feasible for use in measurements in hindered environments.

#### 3.3.3. Hazy Environment

We performed thermal imager measurements under cloudy, foggy, and high-moisture conditions. A hazy environment, unlike a hindered environment, is unavoidable, and haze cannot be eliminated. It represents, for instance, the condition in which moving water particles, acting as obstacles, interfere with thermal radiation because of refraction or their low temperature. We immersed dry ice (approximately −79 °C) in hot water to generate an enormous amount of mist, as shown in Figure 4c. To cover the frame uniformly with mist, we used a minifan to obtain a temperature of 17 °C with high humidity in a hazy environment. Examples of the measured datasets are shown in Figure 5e,f; the error in the average first natural frequencies and average MAC of the mode shapes were 3.84% and 0.9924, respectively (Table 1). Similar with the results under a hindered environment, the low deviation of the data indicates acceptable reliability of the thermal imager measurement under a hazy environment. Even though water particles block the heat radiation, the density of the moving particles is not high enough to interfere with the imager after image processing is performed. The favorable agreement between the accelerometer and thermal imager measurement results demonstrated that the proposed thermal monitoring method is suitable for performing measurements under foggy and high-moisture conditions.

### 3.4. Comparison among Various Environments

From Table 1, the four average frequencies measured by IRT ($f_{1}^{IRT}$) deviates little from each other. Similar results can be observed for the average frequencies measured by accelerometers ($f_{1}^{Acce}$). Both observations indicate that low-visibility conditions hardly affect the measurements using the thermal imager or accelerometers. The indication is obvious for the case of measurement by accelerometers since the measurement by accelerometers is nearly independent of the four tested environmental conditions. As for the case of measurement by IRT, the indication supports the advantages of using thermal imager under various harsh environments. Moreover, the standard deviation of the first natural frequencies for the case of measurements by IRT under a dark environment is the greatest among all four tested conditions. However, all four deviation values fall into acceptable range. This issue can be further resolved by performing multiple measurements under a tested condition. In short, the proposed IRT technique is proved to be effective under low-visibility harsh environment.

## 4. Validation of MSRM for Large-Structure Measurement with Satisfied Resolution

### 4.1. Experimental Setup

**Figure 6:**
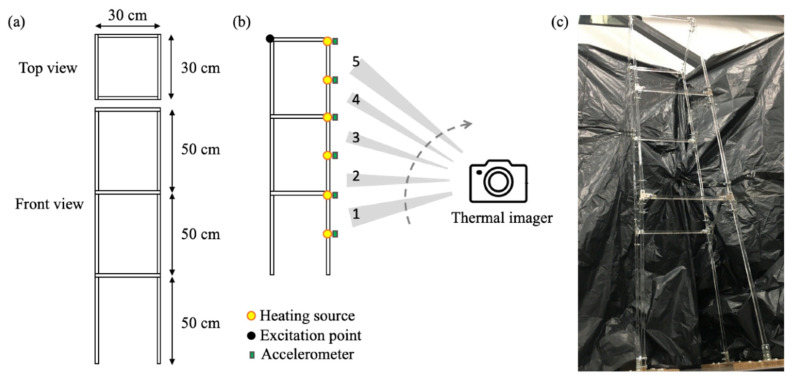
(**a**) 3D acrylic frame structure, (**b**) experimental setup, and (**c**) photo of the entire structure.

Additional 3D acrylic frame structure was used to validate the MSRM. The structure consisted of acrylic beams with a cross-sectional area of 1 mm^2^ × 1 mm^2^ and lengths of 30 and 50 cm; the beams were connected with L-shaped and T-shaped iron pieces, as illustrated in Figure 6a. The 3D frame structure was excited using an impact hammer, and the frame’s vibration responses were measured using six accelerometers and an infrared thermal imager, which was employed to capture the displacement of the six heat sources, as shown in Figure 6b. The connection between the devices in the experiment was the same as that for the experiment described in Section 3.1. The physical model is shown in Figure 6c. Clearly, the details of the structure are not easily identified, and hence the resolution of the images of the structure is expected to be insufficient.

The infrared thermal imager was mounted on a stand, and the camera angle was varied to separate the entire structure into five sections to obtain the corresponding displacement images of the partial structure, as shown in Figure 3b. After image distortion correction, the partial mode shapes of the 3D acrylic frame structure were determined using the methods described in Section 2.3 and Section 2.4. The local mode shapes obtained using the infrared thermal imager were merged into the global mode shape response obtained using the proposed MSRM described in Section 2.6.

### 4.2. MSRM Results

**Figure 7:**
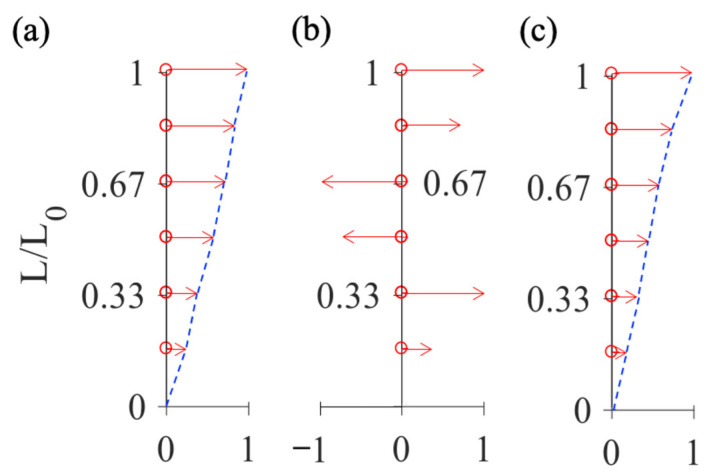
First mode shape result of the 3D frame structure. Mode shape obtained (**a**) with accelerometer data (used as the standard), (**b**) by random recombination of the mode shapes from the thermal imager, and (**c**) by means of the thermal imager and the mode shape recombination method (MSRM) process. (L_0_ = 150 cm).

The mode shape results obtained using the accelerometer datasets were taken as the standard (Figure 7a). The result shown in Figure 7a, which is the first bending mode shape of the 3D frame structure, was used to validate the efficacy of the MSRM. In other words, we verified whether the use of the MSRM on the thermal imaging results is effective. The calculated MAC between the thermal imager data without the MSRM process and the accelerometer data is extremely low, that is, the mode shapes between them are almost independent. The reason for the low MAC is that the random combination, without the MSRM process, cannot guarantee consistency in the vibration phases between different partial mode shapes. For instance, Figure 7b shows that combination of the mode shapes without the MSRM process causes a large phase error, resulting in a low MAC of 0.0645. This indicates that the thermal imager data obtained from a large-scale structure and without use of the MSRM are not useful for SHM. After both image distortion correction and the MSRM process were used to align the phases of vibration according to the repeated nodes, the results of the thermal imager measurements (Figure 7c) were in favorable agreement with the results of the accelerometer measurements (Figure 7a). The calculated MAC between them was 0.9810, indicating that the global response could be obtained correctly when the MSRM was used. Therefore, through the MSRM, the use of an infrared thermal imager for SHM was shown to be effective in measuring not only small-scale and 2D structures but also large-scale and 3D structures.

## 5. Identification of Damage Scenarios under Harsh Environments

**Figure 8:**
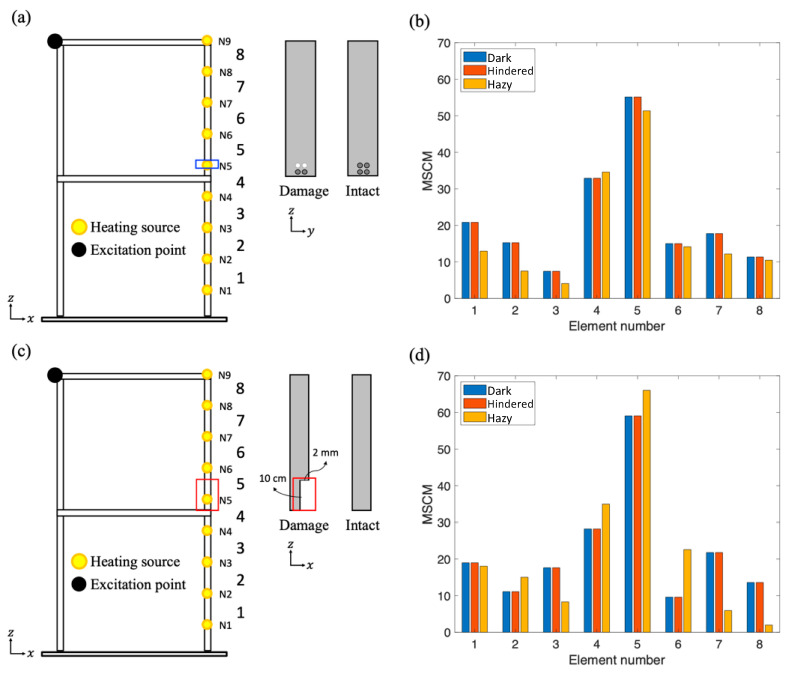
(**a**) Joint damage scenario with damage location (blue box) and schematic showing a comparison of a damaged slat with an intact slat. (**b**) MSCM results for joint damage under various artificial environmental conditions. (**c**) Crack damage scenario with damage location (red box) and schematic showing a comparison of a damaged slat with an intact slat. (**d**) MSCM results of crack damage under various artificial environmental conditions.

The setup used for this experiment was the same as that used for the experiment described in Section 3.1, except that the number of heat sources was nine instead of four for effectively identifying the local damage. The heat sources were linearly placed on one of the vertical columns of the frame structure with 9 cm spacing for the adjacent ones. In Figure 8a, the blue box around node N5 represents joint damage caused by loose screws, and the white points represent the loose screws. Similarly, the 2-mm-thick red box in Figure 8c near elements 4 and 5 represents crack damage. The measurement of the frame structure was performed in dark, hindered, and hazy environments, and the measurement results were processed using the MSRM to locate the damaged area, as shown in Figure 8b,d. The MSCM values (curvature differences) of the joint and crack damage cases (elements 4 and 5) were higher than 27, and they were considerably higher than the values of the other elements, which all had values of lower than 20. The high MSCM values for elements 4 and 5 help identify locations with joint and crack damage.

With regard to the severity of damage, the damaged zone was approximately 10 cm in the crack damage case, whereas the damaged zone in the joint damage case was less than 0.5 cm, which was the diameter of a screw hole. The width of the damaged zone determines the reduction in the stiffness of a slat. If the damage is severe, the curvature of the slat may increase. Therefore, the MSCM values of element 5 in the crack damage case were greater than those in the joint damage case. The results demonstrated that the more severe the damage, the higher the MSCM value. Therefore, the proposed infrared thermography measurement can locate damaged areas and effectively identify the degree of damage, even in various harsh environments.

## 6. Conclusions

In this study, an infrared thermography measurement system was proposed for SHM. The infrared thermal imager was found to be effective in monitoring structures under low-light conditions and harsh environments with a humid atmosphere and obstacles; this is not possible with conventional optical cameras. The feasibility of the infrared thermography method was experimentally investigated by comparing measurements made using infrared thermography with measurements made using accelerometers. The results show that because of the imaging principle of the thermal imager and the image processing process, the vibration signal was obtained even under harsh environments within 3.84% deviation in natural frequency. This proves that the thermal imager is a suitable alternative to the optical camera as a noncontact sensor for SHM.

Moreover, the proposed MSRM, in which partial mode shapes are combined to obtain a complete structural mode shape, helped resolve the issue of insufficient resolution associated with large-scale structural measurements. The findings indicate that infrared thermography is effective in locating and identifying damaged areas on a structure, as well as the severity of damage, under harsh environments when the measured results are processed using the MSCM for the low-frequency vibrational mode.

In conclusion, the proposed methods help overcome many limitations associated with existing SHM methods in harsh or remote environments. Moreover, the use of solar-powered heat sources as sensors for large-scale structural measurement helps overcome the problems associated with traditional sensors, namely, their high cost, long installation time, and tendency to wear out. These sensors may also damage or get entangled between transmission lines; in addition, their accuracy depends on the humidity, and they require light of a sufficient amplitude to function.

Future works can be focused on the application of practical engineering cases such as SHM for offshore wind turbine structures. For instance, if the imager is installed on the service operation vessel for maintenance work, the motion of the vessel can significantly affect the measurements and, hence, needs to be compensated. Moreover, one can expect additional unwanted environmental interferences to heat radiation in real-world applications, such as wind and volant animals. Further clarification of the aforementioned issues is expected to improve the robustness of the proposed SHM system.

## Figures and Tables

**Table 1:** Comparison of infrared thermography (IRT) and accelerometer (Acce.) measurements under various conditions.

Case	Normal	Dark	Hindered	Hazy
Max $f_{1}^{IRT}$ (Hz)	3.77	3.86	3.58	3.64
Average $f_{1}^{IRT}$ (Hz)	3.65	3.69	3.52	3.59
Min $f_{1}^{IRT}$ (Hz)	3.55	3.44	3.47	3.54
SD $f_{1}^{IRT}$	0.11	0.22	0.06	0.05
Max $f_{1}^{Acce}$ (Hz)	3.80	3.70	3.70	3.80
Average $f_{1}^{Acce}$ (Hz)	3.70	3.67	3.63	3.73
Min $f_{1}^{Acce}$ (Hz)	3.60	3.60	3.50	3.70
SD $f_{1}^{Acce}$	0.10	0.06	0.12	0.06
Error (%)	1.35	0.64	3.12	3.84
Average MAC	0.9963	0.9976	0.9803	0.9924

## References

[1] Mieloszyk M., Ostachowicz W. (2017). An application of Structural Health Monitoring system based on FBG sensors to offshore wind turbine support structure model. Mar. Struct..

[2] Weijtjens W., Verbelen T., De Sitter G., Devriendt C. (2016). Foundation structural health monitoring of an offshore wind turbine—A full-scale case study. Struct. Health Monit..

[3] Liao W.I., Wang J.X., Song G., Gu H., Olmi C., Mo Y.L., Chang K.C., Loh C.H. (2011). Structural health monitoring of concrete columns subjected to seismic excitations using piezoceramic-based sensors. Smart Mater. Struct..

[4] Devriendt C., Magalhães F., Weijtjens W., De Sitter G., Cunha Á., Guillaume P. (2014). Structural health monitoring of offshore wind turbines using automated operational modal analysis. Struct. Health Monit..

[5] Bekas D.G., Sharif-Khodaei Z., Aliabadi M.H. (2018). An innovative diagnostic film for structural health monitoring of metallic and composite structures. Sensors.

[6] Mevissen F., Meo M. (2019). A review of NDT/structural health monitoring techniques for hot gas components in gas turbines. Sensors.

[7] Soman R., Balasubramaniam K., Golestani A., Karpiński M., Malinowski P. (2020). A Two-Step Guided Waves Based Damage Localization Technique Using Optical Fiber Sensors. Sensors.

[8] Poozesh P., Baqersad J., Niezrecki C., Avitabile P., Harvey E., Yarala R. (2017). Large-area photogrammetry based testing of wind turbine blades. Mech. Syst. Signal Process.

[9] Lee J.J., Shinozuka M. (2006). displacement measurement of a flexible bridge using digital image processing techniques. Exp. Mech..

[10] Acikgoz S., DeJong M.J., Soga K. (2018). Sensing dynamic displacements in masonry rail bridges using 2D digital image correlation. Struct. Control Health Monit..

[11] Harvey Jr P.S., Elisha G. (2018). Vision-based vibration monitoring using existing cameras installed within a building. Struct. Control Health Monit..

[12] Omidalizarandi M., Kargoll B., Paffenholz J.A., Neumann I. (2018). Accurate vision-based displacement and vibration analysis of bridge structures by means of an image-assisted total station. Adv. Mech. Eng..

[13] Lydon D., Lydon M., Taylor S., Del Rincon J.M., Hester D., Brownjohn J. (2019). Development and field testing of a vision-based displacement system using a low cost wireless action camera. Mech. Syst. Signal Process.

[14] Ehrhart M., Lienhart W. (2015). Monitoring of civil engineering structures using a state-of-the-art image assisted total station. J. Appl. Geod..

[15] Shang Z., Shen Z. (2018). Multi-point vibration measurement and mode magnification of civil structures using video-based motion processing. Autom. Constr..

[16] Ri S., Numayama T., Saka M., Nanbara K., Kobayashi D. (2012). Noncontact deflection distribution measurement for large-scale structures by advanced image processing technique. Mater. Trans..

[17] Feng D., Feng M.Q. (2017). Experimental validation of cost-effective vision-based structural health monitoring. Mech. Syst. Signal Process..

[18] Shan B., Zheng S., Ou J. (2015). Free vibration monitoring experiment of a stayed-cable model based on stereovision. Measurement.

[19] Yang Y., Yu X.B. (2016). Image analyses for video-based remote structure vibration monitoring system. Front. Struct. Civ. Eng..

[20] Shan B., Yuan W., Wang H., Zuo Z., Li S. (2018). Stereovision monitoring for entire collapse of a three-story frame model under earthquake excitation. Struct. Control Health Monit..

[21] Choi A.J., Han J.H. (2018). Frequency-based damage detection in cantilever beam using vision-based monitoring system with motion magnification technique. J. Intell. Mater. Syst. Struct..

[22] Fioriti V., Roselli I., Tati A., Romano R., De Canio G. (2018). Motion Magnification Analysis for structural monitoring of ancient constructions. Measurement.

[23] Gwashavanhu B., Heyns P.S., Oberholster A.J. (2019). Shape principal component analysis as a targetless photogrammetric technique for condition monitoring of rotating machines. Measurement.

[24] Sarrafi A., Mao Z., Niezrecki C., Poozesh P. (2018). Vibration-based damage detection in wind turbine blades using Phase-based Motion Estimation and motion magnification. J. Sound Vib..

[25] Beberniss T.J., Ehrhardt D.A. (2017). High-speed 3D digital image correlation vibration measurement: Recent advancements and noted limitations. Mech. Syst. Signal Process..

[26] Zhong S., Zhong J., Zhang Q., Maia N. (2017). Quasi-optical coherence vibration tomography technique for damage detection in beam-like structures based on auxiliary mass induced frequency shift. Mech. Syst. Signal Process..

[27] Zhong J., Zhong S., Zhang Q., Zhuang Y., Lu H., Fu X. (2016). Vision-based measurement system for structural vibration monitoring using non-projection quasi-interferogram fringe density enhanced by spectrum correction method. Meas. Sci. Technol..

[28] Kylili A., Fokaides P.A., Christou P., Kalogirou S.A. (2014). Infrared thermography (IRT) applications for building diagnostics: A review. Appl. Energy.

[29] Wang X., Fu Z., Wang Y., Liu R., Chen L. (2019). A Non-Destructive Testing Method for Fault Detection of Substation Grounding Grids. Sensors.

[30] Tzitzilonis V., Malandrakis K., Zanotti Fragonara L., Gonzalez Domingo J.A., Avdelidis N.P., Tsourdos A., Forster K. (2019). Inspection of Aircraft Wing Panels Using Unmanned Aerial Vehicles. Sensors.

[31] Mehdi F., Reza T.M. (2019). A scalarization-based method for multiple part-type scheduling of two-machine robotic systems with non-destructive testing technologies. Iran. J. Oper. Res..

[32] Mian A., Han X., Islam S., Newaz G. (2004). Fatigue damage detection in graphite/epoxy composites using sonic infrared imaging technique. Compos. Sci. Technol..

[33] Guo X., Vavilov V. (2013). Crack detection in aluminum parts by using ultrasound-excited infrared thermography. Infrared Phys. Technol..

[34] Talai S.M., Desai D.A., Heyns P.S. (2017). Comparison of infrared thermography and miniature Deltatron accelerometer sensors in the measurement of structural vibration characteristics. Afr. J. Sci. Technol. Innov. Dev..

[35] Talai S.M., Desai D.A., Heyns P.S. (2016). Vibration characteristics measurement of beam-like structures using infrared thermography. Infrared Phys. Technol..

[36] Talai S.M., Desai D.A., Heyns P.S. (2017). Experimentally validated structural vibration frequencies’ prediction from frictional temperature signatures using numerical simulation: A case of laced cantilever beam-like structures. Adv. Mech. Eng..

[37] Ye X.W., Yi T.H., Dong C.Z., Liu T. (2016). Vision-based structural displacement measurement: System performance evaluation and influence factor analysis. Measurement..

[38] Otsu N. (1979). A threshold selection method from gray-level histograms. IEEE Trans. Syst. Man Cybern. Syst..

[39] Haralick R.M., Linda G.S. (1992). Computer and Robot Vision. Vol. 1.

[40] Brincker R., Zhang L., Andersen P. (2001). Modal identification of output-only systems using frequency domain decomposition. Smart Mater. Struct..

[41] Brincker R., Zhang L., Andersen P. Modal identification from ambient responses using frequency domain decomposition. Proceedings of the 18th International Modal Analysis Conference (IMAC 18).

[42] Gade S., Møller N.B., Herlufsen H., Konstantin-Hansen H. Frequency domain techniques for operational modal analysis. Proceedings of the 1st International Operational Modal Analysis Conference (IOMAC 2005).

[43] Allemang R.J. (2003). The modal assurance criterion–twenty years of use and abuse. Sound Vib..

[44] Mahato S., Teja M.V., Chakraborty A. (2017). Combined wavelet–Hilbert transform-based modal identification of road bridge using vehicular excitation. J. Civ. Struct. Health.

[45] Frigui F., Faye J.P., Martin C., Dalverny O., Pérès F., Judenherc S. (2018). Global methodology for damage detection and localization in civil engineering structures. Eng. Struct..

[46] Feng D., Feng M.Q. (2018). Computer vision for SHM of civil infrastructure: From dynamic response measurement to damage detection—A review. Eng. Struct..

[47] He J.H., Liu D.P., Chung C.H., Huang H.H. (2020). Identification of Multiple Local Damage to an Offshore Jacket Substructure Using a Novel Strain Expansion–Reduction Approach. Appl. Sci..

